# Risk factors for late HIV diagnosis in England, 2015–2023

**DOI:** 10.1111/hiv.70156

**Published:** 2025-12-18

**Authors:** Joseph Jasperse, Ross J. Harris, Clare Humphreys, Veronique Martin, Cuong Chau, Alison Brown, Tamara Djuretic, Gareth J. Hughes

**Affiliations:** ^1^ UK Field Epidemiology Training Programme UK Health Security Agency Newcastle upon Tyne UK; ^2^ Field Service North East and Yorkshire and Humber UK Health Security Agency Newcastle upon Tyne UK; ^3^ Statistics Unit UK Health Security Agency London UK; ^4^ South East Health Protection Team UK Health Security Agency Chilton UK; ^5^ HIV Section UK Health Security Agency London UK; ^6^ Field Service North East and Yorkshire and Humber UK Health Security Agency Leeds UK

**Keywords:** HIV, HIV testing, late diagnosis, late presentation, risk factors

## Abstract

**Objectives:**

Late HIV diagnosis increases the risk of premature mortality and onward transmission. We analyzed the risk factors for late diagnosis in England to inform future testing strategies.

**Methods:**

We extracted data from the national HIV surveillance system on all new HIV diagnoses in England residents aged 15 years and older reported between 2015 and 2023 and classified as likely acquiring HIV via sexual transmission. Late diagnosis was defined as a CD4 count <350 cells/mm^3^ within 91 days of diagnosis and no evidence of recent infection. Using multivariable logistic regression, we estimated associations between late diagnosis and age, gender, ethnicity, deprivation, rurality, region of residence, birth in the United Kingdom, likely route of exposure and diagnosis year.

**Results:**

A total of 18 217 diagnoses were included, of which 7177 (39%) were late. Key time‐invariant risk factors for late diagnosis included older age (adjusted odds ratio: 1.44 per 10 years, 95% confidence interval: 1.40–1.49) and, for individuals born outside the United Kingdom, Asian (aOR: 1.69, 95% CI: 1.44–1.98) and Black African (aOR: 1.43, 95% CI: 1.28–1.61) ethnicity. The predicted probability of late diagnosis in men likely exposed via sex with men increased from 21% in 2015 to 32% in 2023, although it was consistently lower than individuals likely exposed through sex between men and women.

**Conclusion:**

Targeted and accessible HIV testing approaches, which extend beyond sexual health services, should be prioritized for older adults, individuals exposed via sex between men and women, and non‐UK‐born ethnic minority groups.

## INTRODUCTION

Late diagnosis of HIV remains a persistent public health challenge with consequences for both individual and population health. Individuals diagnosed late may respond less favourably to antiretroviral therapy (ART) [1, 2], are more likely to require intensive care admission [3], and have a higher risk of morbidity and premature mortality [4, 5, 6]. At the societal level, late diagnosis increases healthcare costs [6, 7, 8] as well as onward transmission, the risk of which is driven by delayed initiation of ART [9] and a missed opportunity to adopt safer sexual practices among individuals unaware of their HIV status [10].

In 2021, the UK government published the HIV Action Plan for England which formalized its commitment to ending new HIV transmissions in England by 2030 [11]. Reducing both the number and proportion of late diagnoses will be essential to achieving this goal and is an important metric for monitoring the effectiveness of testing services. Among individuals first diagnosed in England, the proportion of late diagnoses rose from 35% in 2016 to 40% in 2023 [12, 13]. Following a temporary decline during the COVID‐19 pandemic, the number of late diagnoses has also increased and was higher in 2023 than in 2019 [13]. Modelling estimates meanwhile suggest that 4700 adults were living with undiagnosed HIV infection in England in 2023, equivalent to 5% of the total number of English adults living with HIV [14, 15].

Despite its clinical and public health importance, studies of risk factors for late HIV diagnosis in the United Kingdom remain limited. Previous UK‐based studies have highlighted associations between a higher likelihood of late diagnosis and factors such as older age, sex between men and women, birth outside of the United Kingdom and identification with specific ethnic minority groups [16, 17, 18]. While useful, the generalizability of these studies is limited by their subnational scope and use of an older definition for late diagnosis [19] that did not account for evidence of recent infection and was shown to differentially overestimate late diagnosis in certain groups [20].

This study builds on previous research by conducting multivariable analysis on a national dataset to identify risk factors for the updated definition of late diagnosis in order to inform recommendations for future HIV testing strategies.

## METHODS

### Study population

We conducted a retrospective observational study to identify the risk factors for late HIV diagnosis in England. The study population included English residents aged 15 years and older who were first diagnosed with HIV in the United Kingdom from January 2015 through December 2023 and were reported to the national HIV and AIDS New Diagnoses and Deaths Database maintained by the UK Health Security Agency [21].

### Outcome

Late HIV diagnosis was defined as a CD4 count <350 cells/mm^3^ [3] within 91 days of first diagnosis in the United Kingdom, excluding those with evidence of recent seroconversion [20]. Evidence of recent seroconversion could include either a negative HIV test in the 24 months prior to diagnosis or a result from a recent infection testing algorithm indicating recent infection [13]. Exclusion based on evidence of recent infection was not incorporated in the original European consensus definition of late diagnosis [19], however it is necessary to avoid the misclassification of late diagnosis in individuals who are diagnosed during the seroconversion phase, when a transient reduction in CD4 levels may occur [20, 22, 23].

### Data analysis

We used logistic regression to calculate crude and adjusted odds ratios (aORs) for the binary outcome of late diagnosis and the following variables: year of diagnosis, age at diagnosis, gender identity, ethnic group, whether born in the United Kingdom, index of multiple deprivation (IMD) quintile, rurality, region of residence and probable route of exposure. Age was included as a continuous variable after verifying linearity with the log odds of late diagnosis. Gender was self‐reported by the patient. IMD quintile and rurality were assigned based on the lower super output area of residence, a census area usually containing 1000 to 3000 residents [24, 25]. Ethnicity used the same categories as national surveillance reports [13]. Analysis was restricted to individuals classified by their clinician as having likely acquired their infection via sexual transmission as this was the primary group of interest and accounts for over 95% of new diagnoses with available data [13]. The multivariable analysis was conducted using complete case data, excluding individuals with missing information for the outcome or any covariates.

The above variables were selected a priori by the authors and included in the multivariable model irrespective of their statistical significance. A pre‐specified list of plausible interaction terms was also considered for inclusion in the model. An interaction term was retained in the final model if it satisfied three criteria: (1) the likelihood ratio test indicated statistical significance (*p* < 0.05), (2) the model including the interaction had a lower Akaike information criterion value than the corresponding model without it and (3) visual inspection of the interaction plot suggested that its inclusion materially altered the interpretation of the main effects. Where possible, the variables included in an interaction term were parameterized into a single predictor to aid interpretation. Predicted probability plots and pairwise testing of marginal estimates were used to investigate the relationship between variables included in interaction terms. The final model was further fitted with a random intercept for reporting clinic to account for potential clustering. All analyses were conducted using R version 4.3.1.

### Sensitivity analysis

We performed two sensitivity analyses. First, to investigate whether any association between being born outside the United Kingdom and late diagnosis was driven by infections acquired before arrival in the United Kingdom, we refitted the final model separately on three subsets of data that restricted the inclusion of non‐UK‐born individuals by the duration between their year of arrival in the United Kingdom and year of diagnosis (<5 years, ≥5 years and ≥ 10 years). Secondly, to assess the robustness of our findings to missing data, we conducted multiple imputation of missing explanatory variables using substantive‐model compatible fully conditional specification. This approach uses rejection sampling to impute covariates from imputation models which are compatible with the substantive analysis model, inclusive of any interactions [26]. We then refitted models containing the above fixed effects to 10 imputed datasets, pooled the results using Rubin's rules and compared the pooled results to those from the original (complete case) model [27].

## RESULTS

### Study population

**FIGURE 1:**
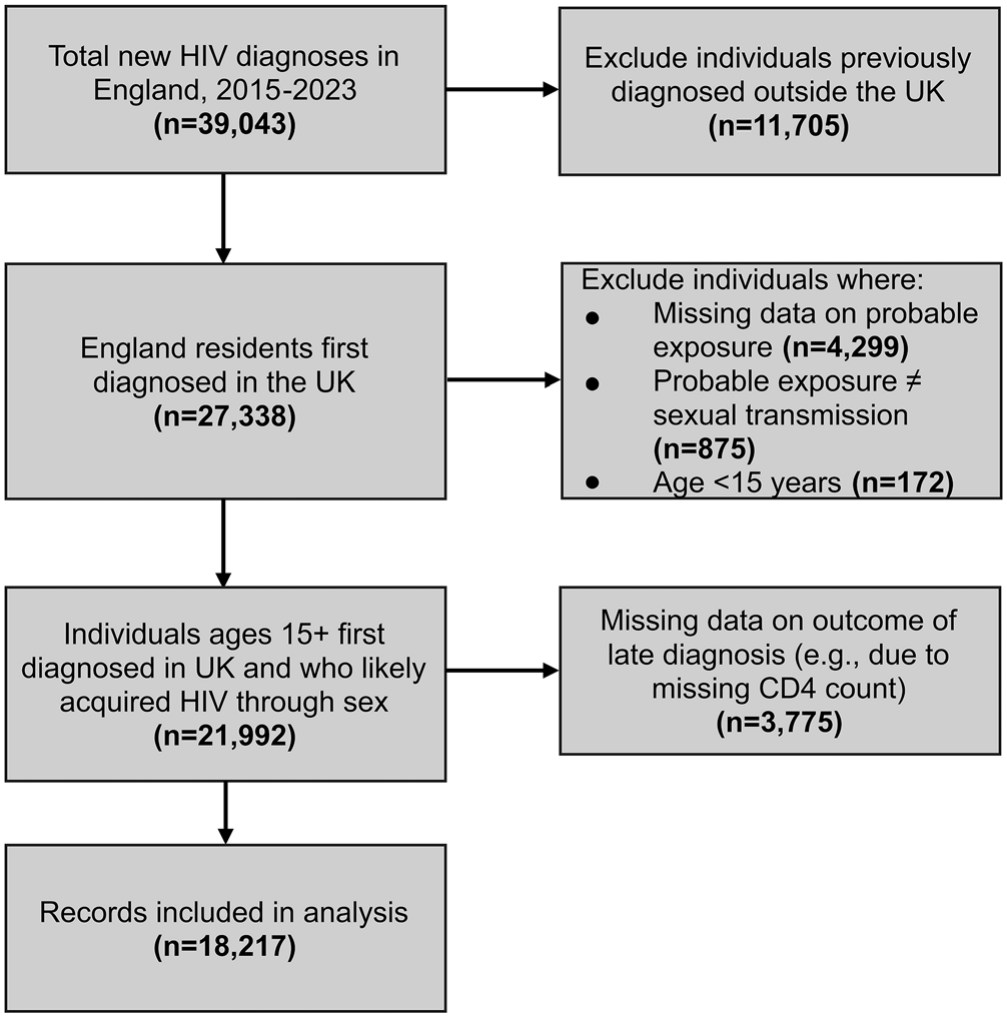
Flowchart of records included in the study analysis.

A total of 18 217 diagnoses were included in the analysis, representing 67% of the 27 338 individuals who were first diagnosed in the United Kingdom during the study period (Figure 1).

The study population is summarized in Table 1. Late diagnoses comprised 39% (7177/18217) of all diagnoses over the study period. Three‐quarters of diagnoses occurred in men, of whom 71% likely acquired HIV through sex with a man. Birth outside the United Kingdom, Black African ethnicity and likely exposure to HIV through sex between men and women were all more commonly reported among individuals who were diagnosed late. Residence outside London accounted for two‐thirds of late diagnoses but less than 60% of non‐late diagnoses.

**TABLE 1:** Number and proportion of late and non‐late HIV diagnoses by potential risk factors, England, 2015–2023.

Characteristic^a^	Entire study population (*n* = 18 217)	Non‐late HIV diagnoses (*n* = 11 040)	Late HIV diagnoses (*n* = 7177)	*p*‐value^b^
Age (median, IQR)	37 (29, 47)	34 (27, 43)	42 (33, 52)	***
Gender				
Man	13 653 (75%)	8682 (79%)	4971 (69%)	***
Woman	4554 (25%)	2349 (21%)	2205 (31%)	
Missing	10 (0%)	9 (0%)	1 (0%)	
Ethnicity				
Asian	1246 (7%)	708 (6%)	538 (7%)	***
Black African	3666 (20%)	1684 (15%)	1982 (28%)	
Black Caribbean	527 (3%)	319 (3%)	208 (3%)	
Black other	411 (2%)	238 (2%)	173 (2%)	
Other/mixed	1578 (9%)	1064 (10%)	514 (7%)	
White	9944 (55%)	6490 (59%)	3454 (48%)	
Missing	845 (5%)	537 (5%)	308 (4%)	
Born in the UK				
Yes	8037 (44%)	5074 (46%)	2963 (41%)	***
No	9125 (50%)	5255 (48%)	3870 (54%)	
Missing	1055 (6%)	711 (6%)	344 (5%)	
Region of residence				
East Midlands	1002 (6%)	556 (5%)	446 (6%)	***
East of England	1607 (9%)	835 (8%)	772 (11%)	
London	6894 (38%)	4561 (41%)	2333 (33%)	
North East	545 (3%)	341 (3%)	204 (3%)	
North West	2027 (11%)	1245 (11%)	782 (11%)	
South East	2141 (12%)	1222 (11%)	919 (13%)	
South West	1065 (6%)	591 (5%)	474 (7%)	
West Midlands	1701 (9%)	963 (9%)	738 (10%)	
Yorkshire and Humber	1235 (7%)	726 (7%)	509 (7%)	
IMD quintile				
5 (least deprived)	1401 (8%)	812 (7%)	589 (8%)	*
4	2047 (11%)	1199 (11%)	848 (12%)	
3	3044 (17%)	1834 (17%)	1210 (17%)	
2	4709 (26%)	2875 (26%)	1834 (26%)	
1 (most deprived)	5072 (28%)	2961 (27%)	2111 (29%)	
Missing	1944 (11%)	1359 (12%)	585 (8%)	
Rural/urban classification				
Rural	999 (5%)	528 (5%)	471 (7%)	***
Urban	15 274 (84%)	9153 (83%)	6121 (85%)	
Missing	1944 (11%)	1359 (12%)	585 (8%)	
Probable exposure route				
Sex between men	9659 (53%)	7020 (64%)	2639 (37%)	***
Sex between men and women	8558 (47%)	4020 (36%)	4538 (63%)	
Year of diagnosis				
2015	3236 (18%)	2173 (20%)	1063 (15%)	***
2016	2662 (15%)	1704 (15%)	958 (13%)	
2017	2220 (12%)	1335 (12%)	885 (12%)	
2018	2100 (12%)	1234 (11%)	866 (12%)	
2019	1969 (11%)	1191 (11%)	778 (11%)	
2020	1302 (7%)	750 (7%)	552 (8%)	
2021	1334 (7%)	709 (6%)	625 (9%)	
2022	1534 (8%)	827 (7%)	707 (10%)	
2023	1860 (10%)	1117 (10%)	743 (10%)	

^a^ Reported as *n* (%) unless otherwise specified. Percentages may not add up to 100% due to rounding.

^b^ Mann–Whitney U test for age. Chi‐squared test for all other variables.

* *p* < 0.05;

*** *p* < 0.001.

### Logistic regression analysis

The main effects from the logistic regression analysis are shown in Table 2. Age remained a significant risk factor for late diagnosis after controlling for other factors (aOR: 1.44 per 10 years, 95% CI: 1.40–1.49). Non‐UK‐born individuals of Asian (aOR: 1.69, 95% CI: 1.44–1.98) and Black African (aOR: 1.43, 95% CI: 1.28–1.61) ethnicities had a higher likelihood of late diagnosis than White individuals born in the United Kingdom in the final model, while UK‐born individuals of other or mixed ethnicities had a lower likelihood (aOR: 0.69, 95% CI: 0.52–0.91). Whereas in univariable analysis the likelihood of late diagnosis was higher in seven of eight regions when compared to London, only the East of England (aOR: 1.39, 95% CI: 1.15–1.69), South East (aOR: 1.23, 95% CI: 1.03–1.46) and West Midlands (aOR: 1.27, 95% CI: 1.03–1.56) remained significantly associated in the multivariable model. There was no clear association between late diagnosis and either IMD quintile or rurality of residence in the multivariable analysis.

**TABLE 2:** Main effects from logistic regression analysis of risk factors for late HIV diagnosis, England, 2015–2023.

Characteristic	Univariable			Multivariable		
	Crude OR	95% CI	*p*‐value	aOR	95% CI	*p*‐value
Age (per 10 years)	1.58	1.54–1.62	***	1.44	1.40–1.49	***
Gender and probable exposure route						
Man, sex with man	Ref			Included as an interaction with year of diagnosis (see Figure 2)		
Man, sex with woman	3.71	3.44–4.01	***			
Woman, sex with man	2.49	2.32–2.68	***			
Ethnicity and birth in the UK						
White, born in the UK	Ref			Ref		
White, not born in the UK	0.69	0.63–0.76	***	1.01	0.90–1.14	0.81
Asian, born in the UK	0.75	0.57–0.99	*	0.82	0.60–1.13	0.22
Asian, not born in the UK	1.45	1.26–1.66	***	1.69	1.44–1.98	***
Black African, born in the UK	1.18	0.88–1.57	0.26	1.12	0.80–1.55	0.52
Black African, not born in UK	2.03	1.87–2.21	***	1.43	1.28–1.61	***
Black Caribbean, born in the UK	0.95	0.73–1.24	0.72	1.00	0.75–1.35	0.98
Black Caribbean, not born in the UK	1.26	0.98–1.63	0.07	0.87	0.64–1.18	0.37
Black other, born in the UK	0.91	0.61–1.33	0.62	0.93	0.61–1.42	0.74
Black other, not born in the UK	1.41	1.10–1.80	**	0.98	0.74–1.30	0.88
Other/mixed, born in the UK	0.59	0.46–0.74	***	0.69	0.52–0.91	**
Other/mixed, not born in the UK	0.93	0.81–1.06	0.25	1.16	0.99–1.36	0.06
Region of residence						
London	Ref			Ref		
East Midlands	1.57	1.37–1.79	***	1.12	0.88–1.42	0.35
East of England	1.81	1.62–2.02	***	1.39	1.15–1.69	***
North East	1.17	0.98–1.40	0.09	0.96	0.71–1.29	0.78
North West	1.23	1.11–1.36	***	1.21	0.99–1.47	0.06
South East	1.47	1.33–1.62	***	1.23	1.03–1.46	*
South West	1.57	1.38–1.79	***	1.22	0.96–1.55	0.10
West Midlands	1.50	1.34–1.67	***	1.27	1.03–1.56	*
Yorkshire and Humber	1.37	1.21–1.55	***	1.01	0.81–1.26	0.94
IMD quintile of residence						
5 (least deprived)	Ref			Ref		
4	0.98	0.85–1.12	0.72	0.98	0.84–1.15	0.81
3	0.91	0.80–1.03	0.15	0.95	0.82–1.10	0.49
2	0.88	0.78–0.99	*	0.98	0.85–1.13	0.77
1 (most deprived)	0.98	0.87–1.11	0.78	0.95	0.82–1.10	0.50
Rural/urban residence						
Urban	Ref			Ref		
Rural	1.33	1.17–1.52	***	1.10	0.94–1.28	0.24
Year of diagnosis						
2015	Ref			Included as an interaction with gender and probable route of exposure (see Figure 2)		
2016	1.15	1.03–1.28	*			
2017	1.36	1.21–1.52	***			
2018	1.43	1.28–1.61	***			
2019	1.34	1.19–1.50	***			
2020	1.50	1.32–1.72	***			
2021	1.80	1.58–2.05	***			
2022	1.75	1.54–1.98	***			
2023	1.36	1.21–1.53	***			

* *p* < 0.05;

** *p* < 0.01;

*** *p* < 0.001.

**FIGURE 2:**
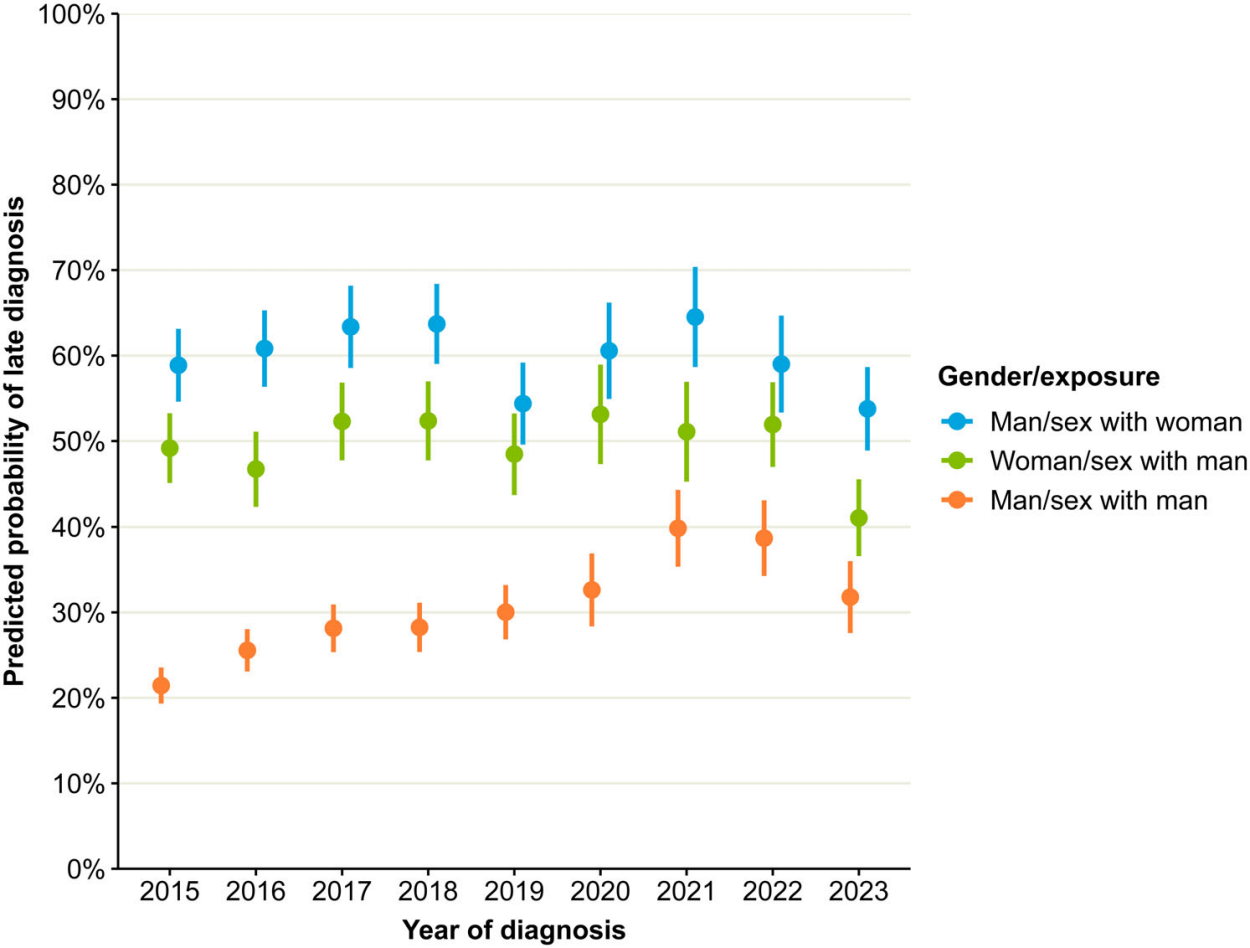
Predicted marginal probability of late HIV diagnosis by gender/probable route of exposure and year of diagnosis, England, 2015–2023.

An interaction term between gender/probable route of exposure and year of diagnosis was included in the final model as it was found to significantly improve model fit even after adjusting for the other covariates (χ^2^ (16) = 44.30, *p* < 0.001). As shown in Figure 2, differences in the predicted probabilities of late diagnosis between the three gender/exposure subgroups narrowed over the study period. Compared to men exposed via sex with men, the predicted probability in men exposed via sex with women was 37% (95% CI: 33%–42%) higher in 2015 but only 22% (95% CI: 16%–28%) higher in 2023. In women exposed via sex with men, the difference reduced from 28% higher (95% CI: 23%–32%) to 9% (95% CI: 3%–15%).

The gradual convergence of these estimates was driven by an increase in the predicted probability of late diagnosis in men exposed via sex with men, which peaked in 2021 but was still 10% higher (95% CI: 6%–15%) in 2023 than in 2015. Women exposed via sex with men meanwhile demonstrated an 8% (95% CI: 2%–14%) decrease over this period. No statistically significant change was evident for men exposed via sex with women.

### Sensitivity analysis

**FIGURE 3:**
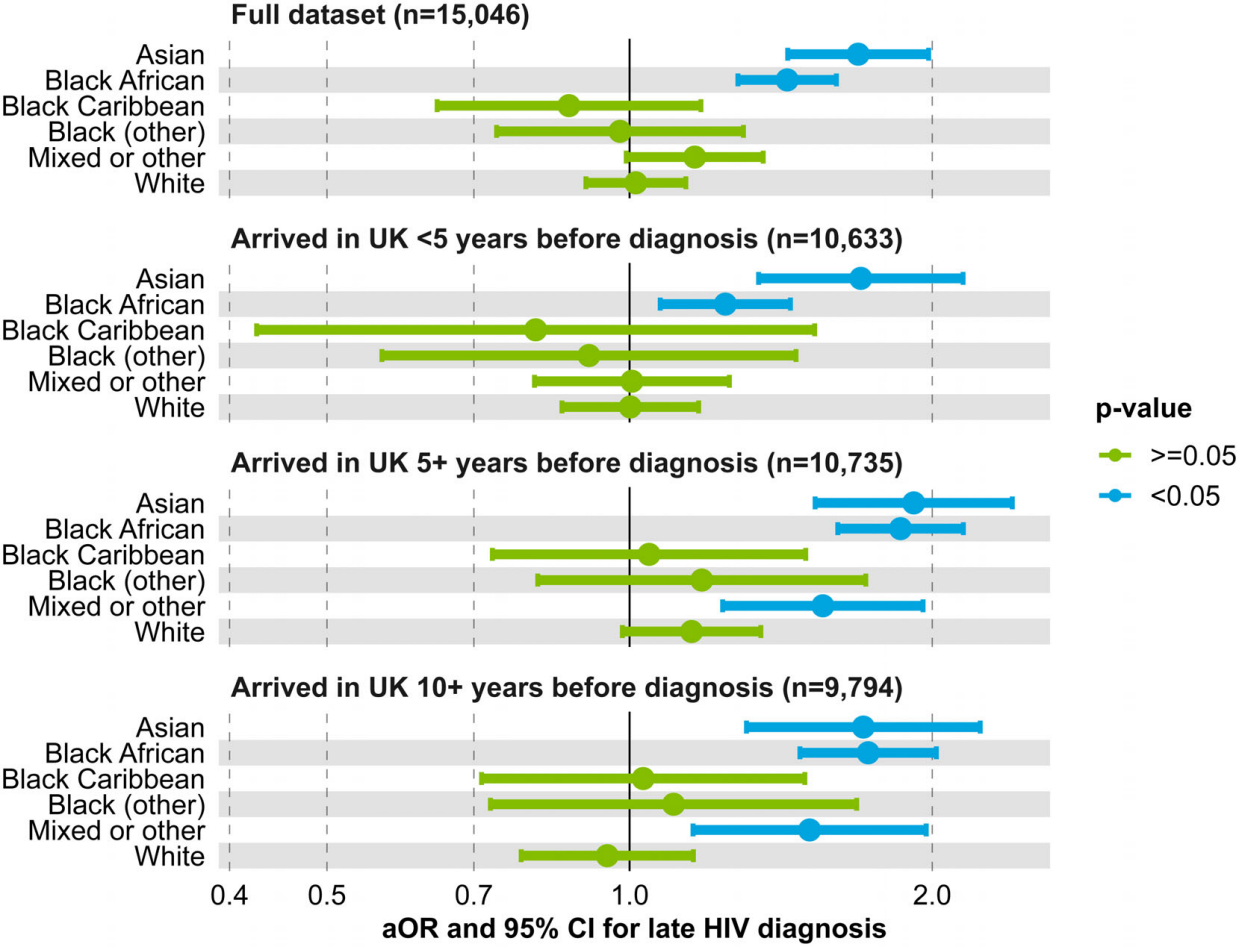
Adjusted ORs and 95% CIs for late HIV diagnosis in non‐UK‐born individuals by ethnicity and duration in the United Kingdom at the time of diagnosis (ref = White, born in the United Kingdom).

Sensitivity analyses indicated that the higher odds of late diagnosis among non‐UK‐born Asian and Black African individuals, relative to UK‐born White individuals, persisted irrespective of duration of residence in the United Kingdom (Figure 3). The association remained robust even in individuals who had arrived in the United Kingdom at least 10 years prior to diagnosis. Individuals of mixed or other ethnicity who had lived in the United Kingdom for at least 5 years at the time of diagnosis were also more likely to be diagnosed late (aOR: 1.56, 95% CI: 1.24–1.96) although this association did not remain significant for the analysis of the full dataset.

A comparison of the final complete case model to the pooled results from multiple imputation is provided in the Supporting Information. All statistically significant risk factors identified in the complete case model remained significant following multiple imputation. In addition, multiple imputation identified significantly higher odds of late diagnosis in all eight regions outside London, whereas the complete case analysis found this only for the East of England, South East and West Midlands.

## DISCUSSION

Our findings confirm and build upon earlier research, identifying non‐UK‐born ethnic minorities (specifically Asian and Black African), older adults, individuals living in selected regions outside of London and people likely exposed to HIV via sex between men and women as groups which are all independently at greater risk of late HIV diagnosis.

The clear association between older age and late diagnosis is consistent with past research both in the United Kingdom and internationally [16, 17, 28, 29, 30]. The reasons for this are likely multifaceted and include reduced perception of HIV risk (among both individuals and healthcare workers), clinician hesitancy to discuss sexual health with older patients, comparatively lower awareness of sexual health services and the added stigma of HIV in older age, which may disincentivize testing [31]. Biological factors, such as the misattribution of early symptoms to the normal effects of ageing and the higher burden of indicator conditions in older adults may likewise contribute to the delayed recognition of HIV in this population [29].

In line with national surveillance data and previous regional studies, we identified a persistently higher odds of late diagnosis in individuals exposed via sex between men and women compared to men exposed via sex with men [13, 16, 17, 18, 30]. This is likely influenced by multiple upstream factors which together contribute to differences in testing, with data from England indicating that the HIV testing rate for gay, bisexual and other men who have sex with men (GBMSM) in sexual health services was 17 times higher than for heterosexual and bisexual women and 28 times higher than for heterosexual men in 2023. Unlike GBMSM, HIV testing rates in heterosexuals remain below their pre‐pandemic levels, underscoring the importance of strengthening testing outside of sexual health services to reduce late diagnosis in this subgroup [13].

Comparisons of the likelihood of late diagnosis over time and across groups are instructive but should be interpreted alongside absolute numbers of new diagnoses. In men exposed via sex with men, the predicted probability for late diagnosis rose from 21% in 2015 to 32% in 2023, concurrent with a 65% reduction in the number of new diagnoses in this subgroup. While changes during the latter half of the study period will be influenced by the disruption caused by the COVID‐19 pandemic, the longer‐term trend may reflect shifts in the composition and risk profile of this population as the pool of undiagnosed individuals shrinks and becomes increasingly comprised of those less likely to regularly test for HIV. Further research exploring changes in the epidemiology and risk factors for late diagnosis in men exposed through sex with men over time would be useful in adapting testing strategies to prevent a plateau in progress. However, this should not detract from efforts to reduce late diagnosis in individuals exposed via sex between men and women, in whom the proportion and number of late diagnoses are both comparatively higher.

The absence of a significant association between late diagnosis and rural residence in the multivariable analysis is notable, especially in light of international research which has identified a link [32, 33, 34] as well as UK studies indicating that individuals in rural areas travel farther to access HIV services and may bypass their local clinic to preserve anonymity [35, 36]. It is possible that the inclusion of a binary rural/urban classification in our model was too crude a measure to detect meaningful differences. Future research would benefit from analysing more direct measures of geographic accessibility, such as travel time to the nearest sexual health clinic, which was unavailable for our analysis.

Consideration of the interaction between ethnicity and birth in the United Kingdom facilitates a more precise understanding of risk factors and is an improvement on past UK studies that have only considered the individual main effects of these characteristics on late diagnosis [16, 17, 18]. Following the combination of ethnicity and birth in the United Kingdom into a single term in our model, only individuals of Asian or Black African ethnicity born outside the United Kingdom had a higher likelihood of late diagnosis than White, UK‐born individuals. The effect persisted in those who arrived in the United Kingdom at least 10 years prior to diagnosis, suggesting that the higher likelihood of late diagnosis in these groups is not attributable solely to pre‐arrival infections but also to differences in testing access and uptake. This finding is consistent with systematic reviews that have identified multiple barriers to HIV testing in migrant populations, including language challenges, low awareness and availability of services, socioeconomic deprivation, racism, stigma and fear regarding the implications of a positive test on immigration status [37, 38, 39]. In our study population, the proportion of new diagnoses among non‐UK‐born individuals rose from 50% in 2015 to 66% in 2023, further reinforcing the need to improve access to culturally sensitive HIV testing services for migrant populations, including long‐term residents.

Our findings are broadly consistent with studies conducted elsewhere in Europe and are likely to be generalizable to other high‐income countries with similar testing infrastructure [40, 41, 42, 43]. Collectively, this growing body of evidence underscores the need for further investment in initiatives that promote earlier HIV diagnosis. For example, indicator condition (IC) guided testing can support earlier diagnosis and is included in testing guidance in the United Kingdom and internationally [44, 45]. While a recent review found suboptimal implementation of IC‐guided testing across Western countries [46], interventions such as clinician training [47], the use of automated testing prompts in electronic health records [48, 49], and the incorporation of HIV testing recommendations into IC‐specific guidelines may help improve adoption [50]. The evidence base for universal opt‐out HIV testing, particularly in hospital emergency departments, is similarly well established [51, 52]. In England, a new opt‐out testing programme across 34 emergency departments identified more than 700 new HIV diagnoses in under 3 years. Individuals diagnosed through the programme were older and more likely to have acquired their infection via sex between men and women compared to those diagnosed in other settings (e.g., sexual health services, primary care) during the same period, highlighting the utility of this approach in reaching populations with different risk factors [53]. These evidence‐based testing approaches complement recent trials on the management of advanced HIV disease, highlighting the need for comprehensive elimination strategies that pair earlier detection with effective treatment for individuals who may still be diagnosed at a late stage of infection [54, 55].

Our analysis is subject to several limitations. First, the exclusion of records with missing data in the primary analysis may have introduced bias. While all risk factors identified in the complete case analysis remained significant in the pooled multiple imputation model, we cannot exclude the possibility that our data are missing not at random. Multiple imputation may also not account for possible bias introduced by records with a missing outcome variable, which were excluded from the pooled imputation model, although the direction and magnitude of such bias are not possible to predict. Second, the restriction of our analysis to infections likely acquired via sexual transmission means the findings may not be generalizable to other exposure routes (e.g., injecting drug use), although these accounted for less than 5% of diagnoses with available data. Third, while the COVID‐19 pandemic may have influenced risk factors, our analysis did not attempt to explicitly model its impact as this was beyond the scope of our primary research question. Nonetheless, this represents a valuable direction for future research.

## CONCLUSION

This study offers the first multivariable analysis of risk factors for late HIV diagnosis in England using a national dataset and updated case definition. Our findings align with previous evidence, demonstrating that individuals who are older, of Asian and Black African ethnicity born outside the United Kingdom, reside in certain regions outside of London or who were likely exposed to HIV through sex between men and women are all at significantly greater risk of late diagnosis. The observed interaction between ethnicity and place of birth suggests that systemic barriers to testing persist even among long‐settled migrant populations. Ultimately, our findings point to the need for more targeted, accessible and inclusive testing strategies that extend beyond sexual health services. Addressing persistent disparities in late diagnosis will be essential to achieving the national HIV Action Plan's target of ending new HIV transmissions in England by 2030 and ensuring that all individuals have timely access to testing, diagnosis and care.

## FUNDING INFORMATION

The study did not require external funding.

## CONFLICT OF INTEREST STATEMENT

The authors have no conflicts of interest to declare.

## ETHICS STATEMENT

Analyses were limited to secondary use of surveillance data routinely collected as part of the public health function of the UK Health Security Agency. A review by a Research Ethics Committee was not required.

## Supporting information


**Data S1.** Supplementary Information.

## Data Availability

Requests for aggregate HIV surveillance data must be submitted in writing to the UK Health Security Agency and are reviewed on a case‐by‐case basis. Further information is available here: https://www.gov.uk/government/publications/hiv-and-aids-reporting-section-hars-data-request-form.
