# Supplementary material for: Risk factors for late HIV diagnosis in England, 2015–2023
Source: HIV Med. 2025 Dec 18;27(3):409–19. doi: 10.1111/hiv.70156 (PMC12968494; doi:10.1111/hiv.70156)
Supplement: Supplementary file 1 — Data S1. Supplementary Information. [file HIV-27-409-s001.docx]

# SUPPORTING INFORMATION

**Supplementary table:** Results of logistic regression analysis of risk factors for late HIV diagnosis using complete case and multiple imputation approaches, England, 2015-2023

| **Characteristic** | **Complete case model** | | | **Multiple imputation model** | | |
| --- | --- | --- | --- | --- | --- | --- |
|  | **aOR** | **95% CI** | **p-value^1^** | **aOR** | **95% CI** | **p-value^1^** |
| **Age (per 10 years)** | 1.44 | 1.40-1.49 | *** | 1.44 | 1.40-1.48 | *** |
| **Gender and probable exposure route** |  |  |  |  |  |  |
| Man, sex with man | Ref |  |  | Ref |  |  |
| Man, sex with woman | 3.12 | 2.49-3.90 | *** | 3.61 | 2.90-4.48 | *** |
| Woman, sex with man | 2.22 | 1.79-2.76 | *** | 2.66 | 2.18-3.23 | *** |
| **Ethnicity and birth in UK** |  |  |  |  |  |  |
| White, born in UK | Ref |  |  | Ref |  |  |
| White, not born in UK | 1.01 | 0.90-1.14 | 0.81 | 0.95 | 0.85-1.05 | 0.30 |
| Asian, born in UK | 0.82 | 0.60-1.13 | 0.22 | 0.98 | 0.74-1.30 | 0.90 |
| Asian, not born in UK | 1.69 | 1.44-1.98 | *** | 1.60 | 1.40-1.83 | *** |
| Black African, born in UK | 1.12 | 0.80-1.55 | 0.52 | 1.08 | 0.80-1.46 | 0.62 |
| Black African, not born in UK | 1.43 | 1.28-1.61 | *** | 1.36 | 1.23-1.51 | *** |
| Black Caribbean, born in UK | 1.00 | 0.75-1.35 | 0.98 | 0.95 | 0.72-1.27 | 0.75 |
| Black Caribbean, not born in UK | 0.87 | 0.64-1.18 | 0.37 | 0.94 | 0.73-1.22 | 0.66 |
| Black other, born in UK | 0.93 | 0.61-1.42 | 0.74 | 0.91 | 0.62-1.34 | 0.64 |
| Black other, not born in UK | 0.98 | 0.74-1.30 | 0.88 | 1.06 | 0.82-1.36 | 0.66 |
| Other/mixed, born in UK | 0.69 | 0.52-0.91 | ** | 0.73 | 0.58-0.93 | * |
| Other/mixed, not born in UK | 1.16 | 0.99-1.36 | 0.06 | 1.07 | 0.94-1.21 | 0.33 |
| **Region of residence** |  |  |  |  |  |  |
| London | Ref |  |  | Ref |  |  |
| East Midlands | 1.12 | 0.88-1.42 | 0.35 | 1.36 | 1.18-1.56 | *** |
| East of England | 1.39 | 1.15-1.69 | *** | 1.43 | 1.27-1.60 | *** |
| North East | 0.96 | 0.71-1.29 | 0.78 | 1.32 | 1.10-1.59 | ** |
| North West | 1.21 | 0.99-1.47 | 0.06 | 1.35 | 1.20-1.51 | *** |
| South East | 1.23 | 1.03-1.46 | * | 1.32 | 1.19-1.47 | *** |
| South West | 1.22 | 0.96-1.55 | 0.10 | 1.48 | 1.29-1.70 | *** |
| West Midlands | 1.27 | 1.03-1.56 | * | 1.28 | 1.14-1.44 | *** |
| Yorkshire and Humber | 1.01 | 0.81-1.26 | 0.94 | 1.30 | 1.14-1.48 | *** |
| **IMD quintile of residence** |  |  |  |  |  |  |
| 5 (least deprived) | Ref |  |  | Ref |  |  |
| 4 | 0.98 | 0.84-1.15 | 0.81 | 0.99 | 0.86-1.14 | 0.88 |
| 3 | 0.95 | 0.82-1.10 | 0.49 | 0.98 | 0.86-1.12 | 0.79 |
| 2 | 0.98 | 0.85-1.13 | 0.77 | 0.95 | 0.83-1.08 | 0.40 |
| 1 (most deprived) | 0.95 | 0.82-1.10 | 0.50 | 0.96 | 0.85-1.09 | 0.55 |
| **Rural/urban residence** |  |  |  |  |  |  |
| Urban | Ref |  |  | Ref |  |  |
| Rural | 1.10 | 0.94-1.28 | 0.24 | 1.11 | 0.96-1.27 | 0.15 |
| **Year of diagnosis** |  |  |  |  |  |  |
| 2015 | Ref |  |  | Ref |  |  |
| 2016 | 1.17 | 0.98-1.40 | 0.08 | 1.15 | 0.98-1.35 | 0.08 |
| 2017 | 1.31 | 1.09-1.57 | ** | 1.41 | 1.20-1.66 | *** |
| 2018 | 1.27 | 1.05-1.53 | * | 1.39 | 1.17-1.65 | *** |
| 2019 | 1.37 | 1.13-1.67 | ** | 1.49 | 1.25-1.77 | *** |
| 2020 | 1.44 | 1.14-1.82 | ** | 1.38 | 1.13-1.69 | ** |
| 2021 | 1.84 | 1.46-2.32 | *** | 1.89 | 1.56-2.28 | *** |
| 2022 | 1.79 | 1.42-2.25 | *** | 1.78 | 1.47-2.14 | *** |
| 2023 | 1.33 | 1.05-1.68 | * | 1.63 | 1.35-1.98 | *** |
| **Year of diagnosis * gender and probable exposure^2^** |  |  |  |  |  |  |
| 2016* Man, sex with woman | 0.89 | 0.64-1.23 | 0.47 | 0.89 | 0.65-1.21 | 0.44 |
| 2017* Man, sex with woman | 0.87 | 0.62-1.22 | 0.42 | 0.78 | 0.57-1.07 | 0.12 |
| 2018* Man, sex with woman | 0.89 | 0.64-1.25 | 0.52 | 0.81 | 0.59-1.12 | 0.20 |
| 2019* Man, sex with woman | 0.57 | 0.41-0.80 | ** | 0.59 | 0.43-0.80 | *** |
| 2020* Man, sex with woman | 0.72 | 0.49-1.06 | 0.09 | 0.71 | 0.50-1.01 | 0.05 |
| 2021* Man, sex with woman | 0.63 | 0.42-0.94 | * | 0.59 | 0.41-0.86 | ** |
| 2022* Man, sex with woman | 0.52 | 0.35-0.76 | *** | 0.56 | 0.41-0.78 | *** |
| 2023* Man, sex with woman | 0.55 | 0.38-0.79 | ** | 0.41 | 0.29-0.57 | *** |
| 2016*Woman, sex with man | 0.76 | 0.56-1.03 | 0.08 | 0.78 | 0.58-1.03 | 0.08 |
| 2017*Woman, sex with man | 0.87 | 0.64-1.19 | 0.39 | 0.75 | 0.57-1.00 | * |
| 2018*Woman, sex with man | 0.88 | 0.64-1.21 | 0.44 | 0.84 | 0.62-1.14 | 0.26 |
| 2019*Woman, sex with man | 0.64 | 0.46-0.89 | ** | 0.62 | 0.46-0.84 | ** |
| 2020*Woman, sex with man | 0.78 | 0.53-1.14 | 0.20 | 0.76 | 0.54-1.07 | 0.11 |
| 2021*Woman, sex with man | 0.51 | 0.35-0.74 | *** | 0.50 | 0.36-0.68 | *** |
| 2022*Woman, sex with man | 0.58 | 0.40-0.82 | ** | 0.51 | 0.38-0.69 | *** |
| 2023*Woman, sex with man | 0.48 | 0.34-0.67 | *** | 0.41 | 0.30-0.55 | *** |
| ^1^ *p<0.05, **p<0.01, ***p<0.001  ^2^ The coefficients for the interaction term should be interpreted relative to the reference categories for year of diagnosis (2015) and gender/exposure (man/sex with man). | | | | | | |
